# Differential responsiveness of MET inhibition in non-small-cell lung cancer with altered CBL

**DOI:** 10.1038/s41598-017-09078-4

**Published:** 2017-08-23

**Authors:** Yi-Hung Carol Tan, Tamara Mirzapoiazova, Brian M. Won, Li Zhu, Minu K. Srivastava, Everett E. Vokes, Aliya N. Husain, Surinder K. Batra, Sherven Sharma, Ravi Salgia

**Affiliations:** 1 Department of Medicine, Section of Hematology/Oncology, The University of Chicago Medicine and Biologic Sciences, Chicago, IL USA; 2 Department of Medical Oncology and Therapeutic Research, City of Hope, Duarte, CA USA; 3 Department of Medicine, University of California Los Angeles, Los Angeles, CA USA; 4 Department of Pathology, The University of Chicago Medicine and Biologic Sciences, Chicago, IL USA; 5 Department of Biochemistry and Molecular Biology, University of Nebraska College of Medicine, Omaha, NE USA; 6 Fred and Pamela Buffett Cancer Center, University of Nebraska Medical Center, Omaha, NE USA

## Abstract

Casitas B-lineage lymphoma (CBL) is an E3 ubiquitin ligase and a molecule of adaptor that we have shown is important for non-small-cell lung cancer (NSCLC). We investigated if MET is a target of CBL and if enhanced in *CBL*-altered NSCLC. We showed that *CBL* wildtype cells have lower MET expression than *CBL* mutant cells. Ubiquitination of MET was also decreased in *CBL* mutant cells compared to wildtype cells. Mutant cells were also more sensitive to *MET* inhibitor SU11274 than wild-type cells. sh-RNA-mediated knockdown of *CBL* enhanced cell motility and colony formation in NSCLC cells, and these activities were inhibited by SU11274. Assessment of the phospho-kinome showed decreased phosphorylation of pathways involving MET, paxillin, EPHA2, and VEGFR. When *CBL* was knocked down in the mutant cell line H1975 (erlotinib-resistant), it became sensitive to MET inhibition. Our findings suggest that *CBL* status is a potential positive indicator for MET-targeted therapeutics in NSCLC.

## Introduction

Lung cancer is the second most common cancer in both men and women, and it has a very poor prognosis^1^. Non-small-cell lung cancer (NSCLC), a type of lung cancer, accounts for 80% of all lung cancers, and it has a 5-year survival rate of approximately 15%^2^. Despite recent developments in targeted therapeutic approaches and immune-therapies, the overall morbidity and mortality of NSCLC have not changed substantially over the past 25 years. Therefore, there is an urgent need to identify and develop novel targeted therapies.

Receptor tyrosine kinases (RTKs) are involved in cell cycle, proliferation, and differentiation in cancer^3, 4^. Multiple studies have shown that RTKs are overexpressed as oncogenes in various cancers including lung cancer^5, 6^. Therefore, targeting RTKs is a new strategy for inhibiting tumor growth^7^. Many studies have indicated that *CBL* (Casitas B-lineage lymphoma) plays an important role in down-regulating RTKs based on its E3 ubiquitin ligase activity^8, 9^. The CBL protein family belongs to a class of E3 ubiquitin ligases^10^. The CBL protein associates with the endocytosis mechanism, and plays a crucial role in terminating RTK signaling^10^. The tyrosine kinase binding (TKB) and RING finger domains of CBL are the crucial domains for regulating RTK signaling, particularly EGFR and MET regulation^7^.

Mutations in *CBL* were first reported in human acute myeloid leukemia (AML), and over the past several years, *CBL* mutations have been identified in other types of leukemia^11, 12^. Our previous studies were the first to report *CBL* mutations in solid tumors, such as lung cancer^13^. Eight novel somatic mutations were found in Caucasian, Taiwanese, and African American patients with NSCLC. Moreover, loss of heterozygosity (LOH) was detected in 22% of NSCLC cases, and none of these patients’ samples had any mutations in their remaining copy of *CBL* ^13^. Of the eight novel *CBL* mutations, three displayed relevant E3 ubiquitin activity; S80N/H94Y, Q249E, and W802*. Ectopic expression of these mutations in NSCLC cell lines enhanced cell proliferation and motility^13^. In contrast, ectopic expression of wild-type (WT) *CBL* inhibited NSCLC cell proliferation *in vitro* and tumor formation *in vivo* ^14^. Interestingly, our previous research showed EGFR expression was similar in *CBL* WT and *CBL* mutant (Mt) cells.

*MET* has been identified as an important target in various human cancers, especially in lung cancer. *MET* signaling plays a critical role in tumor cell survival, proliferation, and migration. *MET* is mutated (juxtamembrane domain) and amplified in 4% and 5%, of lung cancer cases, respectively^15, 16^. In addition, more than 50% of lung cancer patients have MET overexpression^15, 16^. NSCLC patients with *MET* mutations and amplifications, as well as MET overexpression, displayed stronger responses to MET inhibitors^17–19^.

To understand whether the different *CBL* Mts affect the E3 ubiquitin ligase activity, *EGFR* was investigated as a model target for CBL E3 ubiquitin function in our previous experiment^13^. The results showed that all of the CBL Mts had similar ubiquitination of the activated EGFR to the CBL WT protein. The ubiquitination of MET, however, was decreased in A549 cells that transiently expressed CBL Mts relative to CBL WT cells. The preliminary results demonstrated that the substrate of CBL E3 ubiquitin activity was MET but not EGFR. Hence, in the current study, we sought to not only determine if MET is a target for CBL-mediated degradation and ubiquitination in NSCLC, and also whether it could serve as a novel therapeutic target in lung cancer.

## Results

### MET expression is increased in *CBL* mutants and shRNA knockdown cells

**Figure 1:**
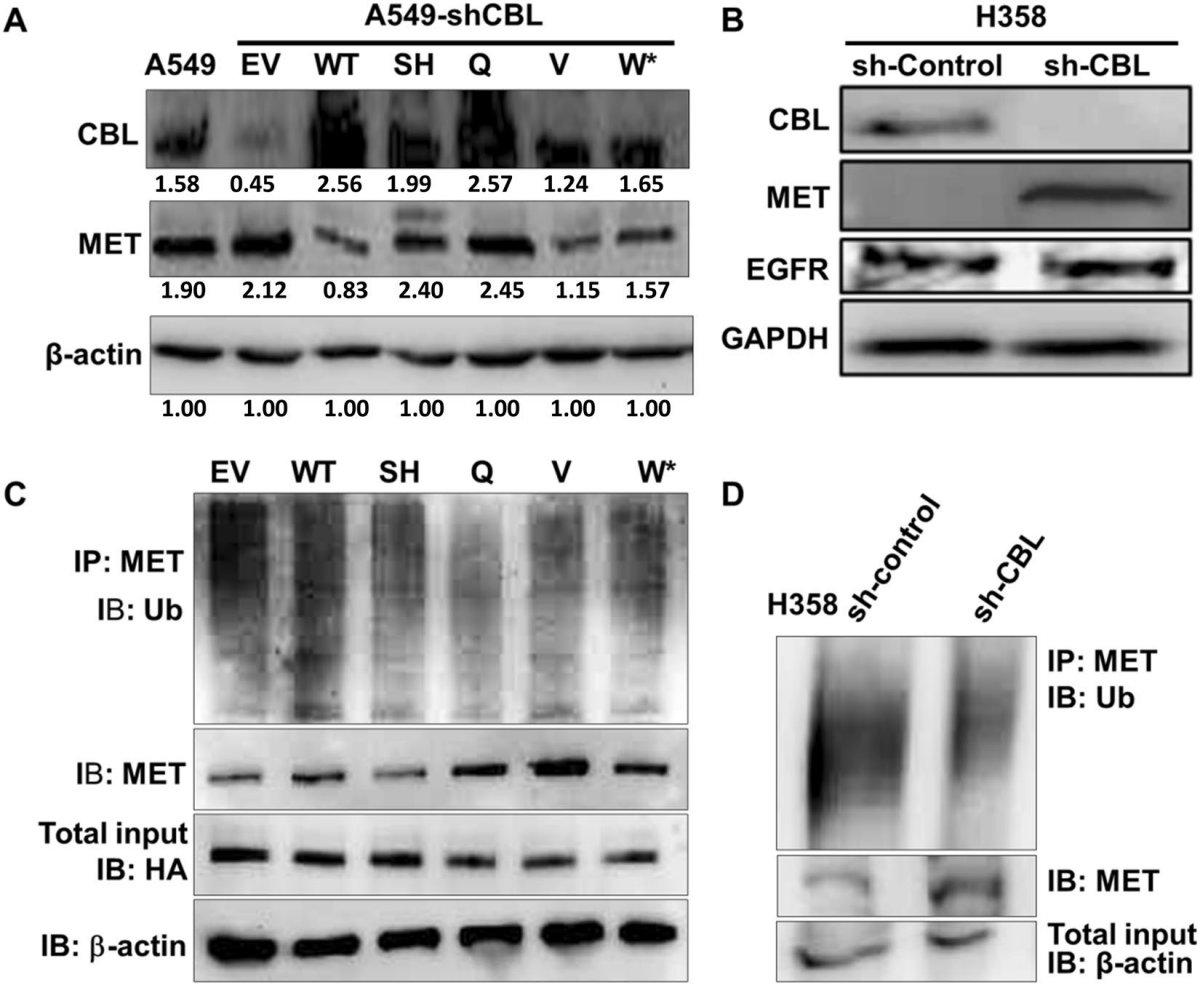
Ubiquitination and expression analysis of various *CBL* mutants. (**A**) A549 shRNA knockdown *CBL* cells were transiently transfected with various *CBL* mutants (SH: S80N/H94Y, Q: Q249E, V: V391I, W*: W802*) and wild-type (WT). MET protein showed low expression in *CBL* WT and high expression in *CBL* Mts isogenic cells. Protein expression was quantified and indicated with the fold change numbers shown below each immunoblot in comparison with loading control β-actin. (**B**) MET and EGFR expression of H358 sh-control and sh-CBL. MET showed higher expression in sh-CBL than sh-control cells. EGFR had no difference in sh-control and sh-CBL. (**C**) A549 cells transiently transfected with empty vector (EV) or *CBL* WT and Mts (SH: S80N/H94Y, Q: Q249E, V: V391I, W*: W802*). Whole cell lysates were IP with anti-MET antibody and IB with anti-Ub antibody. IB with anti-HA antibody for transfection efficiency and β-actin for loading control of the IP. The results showed the ubiquitination of MET were decreased in A549 cells that transiently expressed CBL mutants relative to CBL WT cells. (**D**) H358 sh-Control and sh-CBL cell lysates were IP with anti-MET antibody and IB with anti-Ub antibody β-actin for loading control of the IP. The results showed the ubiquitination of MET were decreased in sh-CBL cells relative to sh-control cells. Each protein lysates of separated blot of were collected in the same time period for and the lysates were loaded in one gel per antibody staining.

To investigate whether *CBL* mutations we identified previously affect the protein expression regulation of both EGFR and MET in NSCLC, we first used anti-*CBL* shRNA to silence *CBL* in A549 cells that had very low CBL endogenous protein expression. We then overexpressed CBL WT and CBL Mts S80N/H94Y, Q249E, V391I, and W802* to make stable clones. Sh-RNA knockdown *CBL* (sh-CBL) in H358 cells that had high CBL expression was also used. MET expression was decreased in A549 *CBL* WT cells and increased in most of A549 *CBL* Mts and H358 sh-CBL knockdown cells by immunoblotting (Fig. 1). A549 *CBL* Mt-S80N/H94Y and Q249E showed significantly increasing MET expression and A549 *CBL* Mt- V391I and W802* showed only slightly higher MET expression compared to A549 *CBL* WT (Fig. 1). However, EGFR protein expression did not show differences in A549 isogenic *CBL* WT or Mt cells^13^ or in H358 shRNA knockdown cells (Fig. 1A and B).

### E3 ubiquitin ligase function of *CBL* mutations and knockdown is decreased in degrading MET

We previously showed that EGFR protein expression had no difference while *CBL* mutations occurred^13^. To investigate whether *CBL* mutations affect the E3 ubiquitin activity, immunoprecipitation followed by immunoblotting showed that ubiquitin decreased in A549 *CBL* Mt and H358 sh-CBL cells compared with A549 *CBL* WT and H358 sh-control cells (Fig. 1C and D). MET protein expression showed higher expression in A549 *CBL* Mt and H358 sh-CBL cells compared to A549 *CBL* WT and H358 sh-control cells.

### *CBL* mutations and knockdown cells increase the sensitivity of SU11274 treatment in cell survival and migration

**Figure 2:**
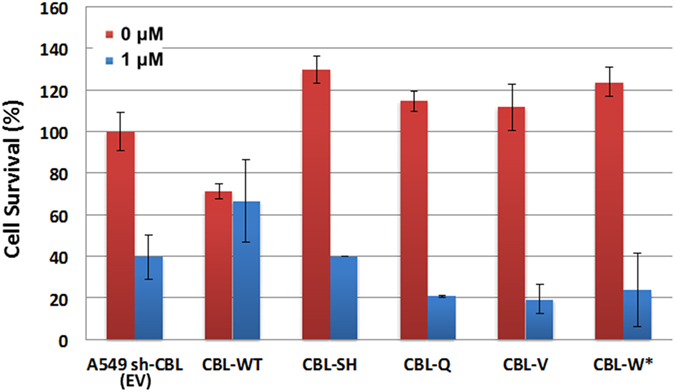
Cell survival of *CBL* Mts after treatment with MET inhibitors. A549 *CBL* isogenic cells with empty vector (EV) or *CBL* wild-type (WT) and mutations (SH: S80N/H94Y, Q: Q249E, V: V391I, W*: W802*). MET inhibitor SU11274 was treated 1 μM for 48 hr. *CBL* Mts showed more sensitivity to SU11274 treatment than *CBL* WT.

Since *CBL* Mt and knockdown cells have higher MET protein expression than *CBL* WT cells, we hypothesized that *CBL* Mt and knockdown cells would be more sensitive to MET inhibitory treatment. To investigate whether overexpression of MET enhanced the sensitivity of drug treatment, cell viability was assayed. Results for cell survival of SU11274 treatment in empty vector (EV) control and CBL Mt cells; S80N/H94Y, Q249E, V391I, and W802*, which have low CBL/high MET expression were 39.8%, 39.9%, 21.1%, 19.3%, and 23.9%, respectively. *CBL* WT cells showed a 66.7% of survival rate after SU11274 treatment (Fig. 2).

**Figure 3:**
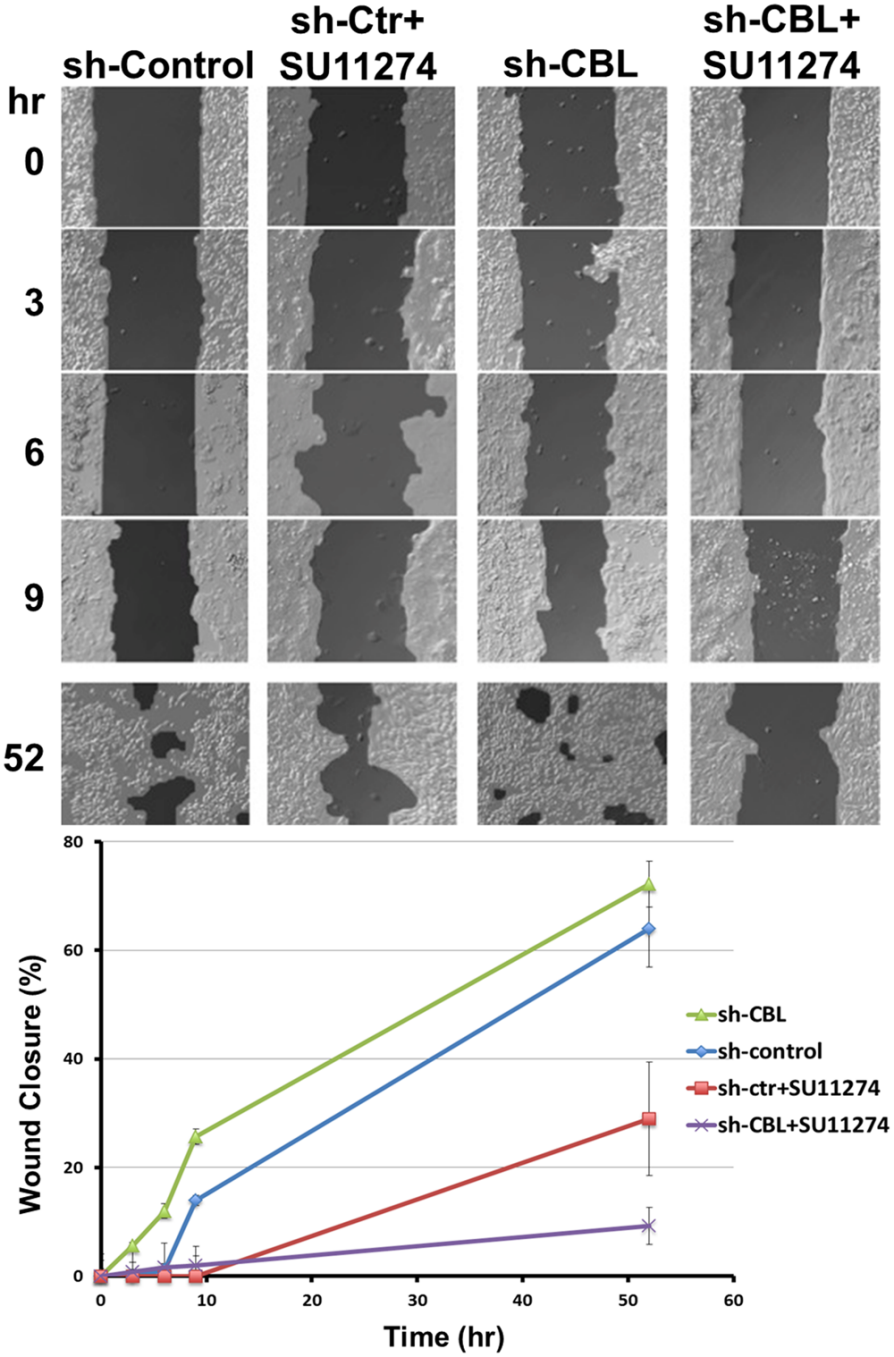
Cell migration of sh-CBL cells treated with MET inhibitor SU11274. Wound healing assay was performed in H358 sh-control and sh-CBL cells. The results showed sh-CBL cells had higher migration ability than sh-Control cells. After treated with MET inhibitor SU11274, sh-CBL cells had more sensitive migration inhibition by SU11274 than sh-control cells.

In addition, we performed a cell migration (wound healing) assay in H358 sh-CBL isogenic cells. The results showed that the wound gap in H358 sh-CBL cells was smaller than the gap in sh-control cells. When treated with MET inhibitor SU11274, sh-CBL cells showed a larger wound gap than the sh-control cells. Thus, H358 sh-CBL cells, which had higher expression of MET compared with sh-control cells, had higher migration ability than the control cells but migration ability decreased after SU11274 treatment (Fig. 3). In summary, cell survival and migration assays both showed that *CBL* Mts and knockdown cells increased the sensitivity of MET inhibitor SU11274.

### *CBL* knockdown cells enhance colony formation and are more sensitive to MET inhibitor SU11274

**Figure 4:**
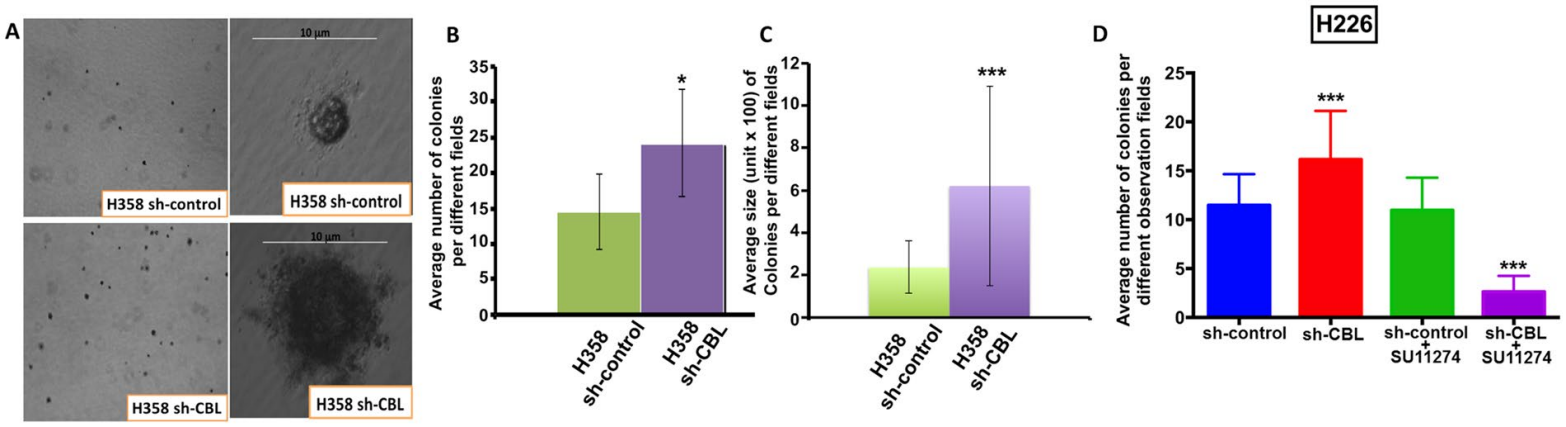
*CBL* knockdown increases colony formation and the cells were sensitive to MET inhibitor SU11274. (**A**) Soft agar colony formation assay showed colony formation of H358 sh-control and sh-CBL cells. sh-CBL cells showed more colonies and larger colony size than sh-control cells. (**B** and **C**) Show the colony number and size quantification respectively. **(D) S**oft agar colony formation assay showed colony formation of sh-CBL cells alone and treated with MET inhibitor SU11274 had significant more colonies and inhibition by SU11274 respectively than sh-control cells. P-values were for comparison with sh-control cells. (*p < 0.05 and ***p < 0.001).

In our previous studies, H358 sh-CBL cells had increased cell proliferation compared with sh-control cells^13^. In addition, *CBL* WT cells had decreased cell and tumor growth^13, 14^. To evaluate *CBL* Mt/knockdown effects in culture, a soft agar colony formation assay was performed by first using H358 sh-CBL cells. The results showed H358 sh-CBL cells had more colony formations and the colony size was larger than sh-control cells (Fig. 4).

H226 sh-CBL cells treated with SU11724 showed a larger number of colonies inhibition than sh-control cells (Fig. 4D).

### CBL mutations affect multi phospho-RTKs on the phospho-kinome

**Figure 5:**
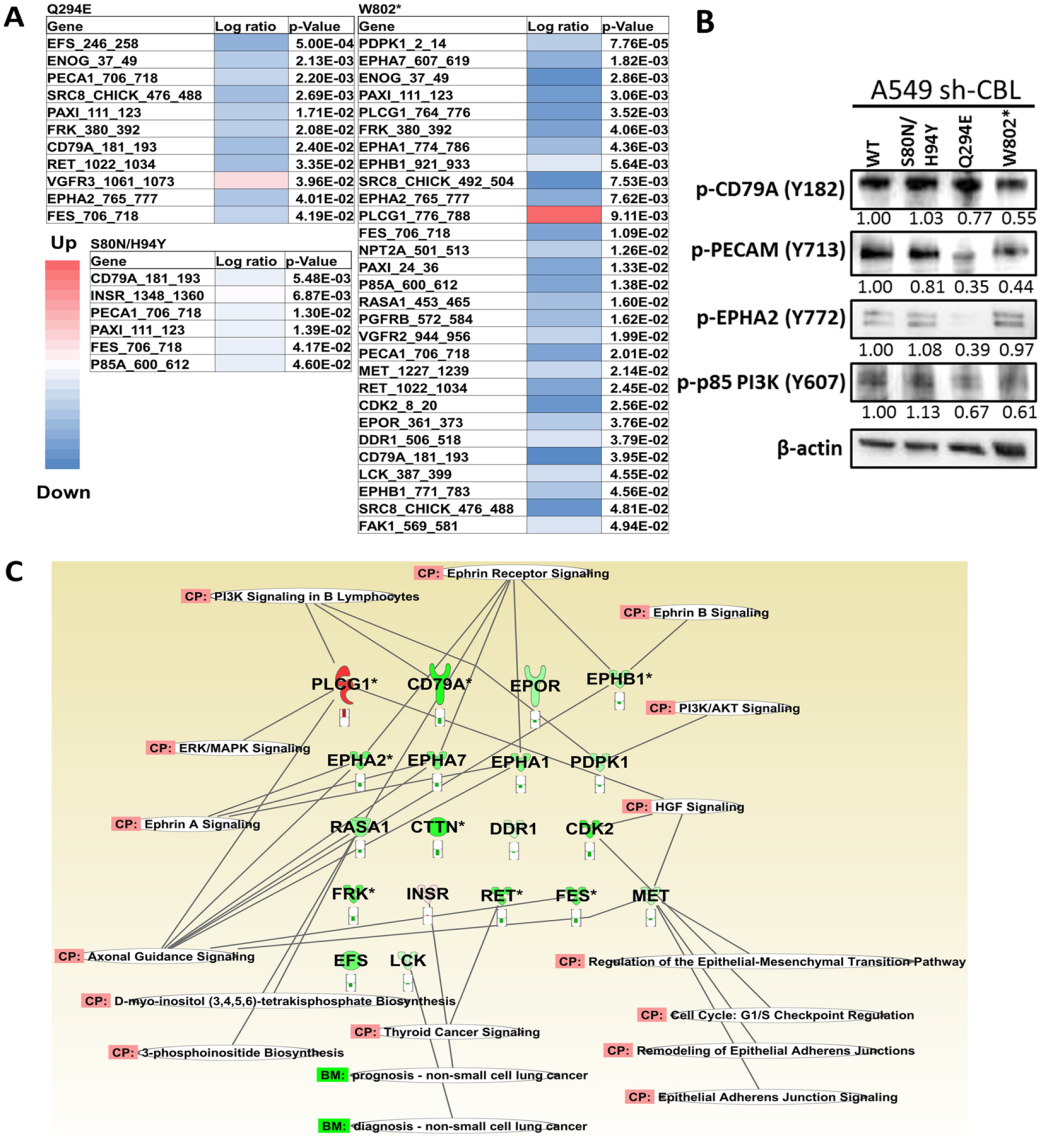
The Heatmap of CBL mutations. (**A**) PamGene analysis was performed to detect the RTK phosphorylation difference between CBL WT and mutants. A549 *CBL* isogenic cells were treated with MET inhibitor SU11274. After treatment with SU11274, and in comparison with CBL WT, significantly changed peptides were shown as a heatmap. Red color represents the signal upregulation and the blue color represents the signal downregulation. (**B**) The protein expression of common targets in three CBL mutations was validated by immunoblotting. Protein expression was quantified and indicated with the fold change numbers shown below each immunoblot in comparison with WT. Each protein lysates of separated blot of were collected in the same time period for and the lysates were loaded in one gel per antibody staining. (**C)** Significantly different peptides were used to analyze affected signaling pathways by Ingenuity Pathway Analysis (IPA). PI3K/AKT, ephrin A, ERK/MAPK, and other signaling pathway were involved especially *MET/HGF* signaling pathway.

We determined the phospho-kinome activity of CBL Mts S80N/H94Y, Q249E, and W802* after SU11274 treatment. The analysis showed the receptor tyrosine kinase activity of most peptides was down regulation, and a total of 32 peptides from 28 genes were significantly modulated (p < 0.05) (Fig. 5A). Three tyrosine kinase phosphorylation sites were significantly detected down regulated in S80N/H94Y, Q249E, and W802* CBL mutants (p < 0.05): CD79A molecule, immunoglobulin-associated alpha also known as B-cell antigen receptor complex-associated protein alpha chain and MB-1 membrane glycoprotein (CD79A) (Tyr86/92); paxillin (PAXI) (Tyr118); and FES proto-oncogene (FES) (Tyr713). The W802* CBL mutation showed the most dramatic tyrosine kinase phosphorylation inhibition. In addition, VEGFR3, also known as fms-related tyrosine kinase 4 (FLT4) (Tyr1063/1068), and phospholipase C gamma 1 (PLCG1) (Tyr783) were the only two sites found to be significantly upregulated (p < 0.05) (Fig. 5A). The protein expression was selectively validated by immunoblotting. p-CD79A, p-PECAM, p-EPHA2, and p-p85 PI3K were all found significantly different than CBL WT in all three mutants (Fig. 5B).

To understand the network of affected peptides, a signaling transduction network with the 32 down-regulated peptides was predicted and created by IPA (Fig. 5C). The analysis revealed that *PTEN* signaling was the top canonical pathway (p < 0.001) involved in MET inhibition in *CBL* Mt cells. In addition, carbohydrate metabolism, cellular function/maintenance, and cell-to-cell signaling/interaction were the networks which had the most affected peptides involved. Moreover, the genes of the most affected peptides are involved in cell cycle, cell death, and cell survival related to cancer and respiratory disease (Table 1).

**Table 1:** Summary of PamGene IPA analysis.

**Top Canonical Pathway**	**p-value**
PTEN Signaling	1.06E-04
Axonal Guidance Signaling	1.8E-04
Semaphorin Signaling in Neurons	9.7E-04
GDNF Family Ligand-receptor Interactions	1.29E-03
STAT3 Pathway	1.44E-03
**Top Diseases and Bio Functions**	
**Diseases and Disorders**	**p-value**
Cancer	2.06E-03
Respiratory Disease	2.06E-03
**Molecular and Cellular functions**	**p-value**
Cell Cycle	1.03E-03
Cell Death and Survival	2.06E-03
Cellular Compromise	2.06E-03
Cellular Response to Therapeutics	2.06E-03
Cellular Development	1.23E-02
**Top Networks**	
**Associated Network Function**	Score*
Carbohydrate metabolism, Cellular Function and Maintenance, Cell-To-Cell Signaling and Interaction	2
Cell Morphology, Hematological System Development and Function, Inflammatory Response	2
Cellular Movement, Cell Death and Survival, Cancer	2
Cell Cycle, DNA Replication, Recombination, and Repair, Cancer	1

*The score of top networks is simply a measure of the number of genes significantly different from CBL WT or Mts in a network.

### *EGFR* mutant/*MET* wild-type cells with *CBL* knockdown enhance the sensitivity of inhibition by MET inhibitor SU11274

**Figure 6:**
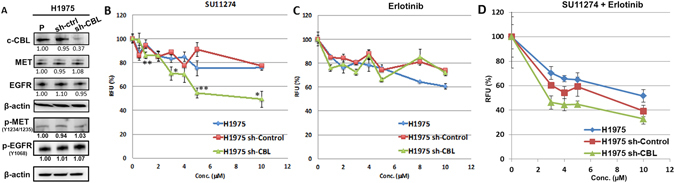
MET and EGFR inhibition in *EGFR* mutation cell line H1975. **(A)** CBL, MET, p-MET, EGFR, and p-EGFR protein expression in H1975 CBL knockdown cells. Protein expression was quantified and indicated with the fold change numbers shown below each immunoblot in comparison with parental H1975 cells. Each protein lysates of separated blot of were collected in the same time period for and the lysates were loaded in one gel per antibody staining. (p: parental, c: sh-control). **(B)** MET inhibitor SU11274, **(C)** EGFR inhibitor erlotinib, and **(D)** SU11274 and erlotinib combination were used to treat H1975, H1975 sh-control, and H1975 sh-CBL cells with variant dosages for 24 hr. *p < 0.05, **p < 0.01. RFU: relative fluorescence units.

Using the University of Chicago Thoracic Oncology Research Program (TORP) database, we found patients with *CBL* alterations also had other driver gene alterations. Table 2 lists *MET*, *EGFR*, or *KRAS* alterations of each patient who had a *CBL* alteration and the treatment outcome. Most patients with *CBL* alterations had *EGFR* mutations but not *MET* mutations. Also, most patients with *CBL* alterations and *EGFR* mutations had a poor response to the treatment. Hence, to investigate the finding of clinical results and whether *CBL* could be a positive indicator for *MET*-targeted therapeutics in lung cancer, we used the *EGFR* L858R/T790M mutation cell line H1975. H1975 cells have WT *MET*. H1975 sh-CBL cells remained resistant to the EGFR inhibitor, erlotinib (Fig. 6). However, H1975 sh-CBL cells became sensitive to SU11274, a MET inhibitor, compared to sh-control and parental H1975 cells (Fig. 6).

**Table 2:** Foundation ONE reports of CBL alterations with EGFR, MET, or KRAS alterations and patient treatment outcome.

Patient	Tumor type	*CBL* mutation	*EGFR/MET/KRAS* mutation	Chemo/Clinical Trial	Outcome
1	Lung AD	H37_H38insHH	***EGFR*** E746_A750del, T790M	Erlotinib	Was effective for a time but progressive disease
				AP26113	1. Interval progression of disease with interval increase in the size of the left lung mass, hepatic metastases, also as metastases and left adrenal metastatic disease.
					2. Interval development of right-sided pulmonary static disease.
2	Lung AD	A848T	***EGFR*** E746_A750del, amplification	Carboplatin	Unknown. Tolerated Well
				Paclitaxel	Unknown. Tolerated Well
				Erlotinib	Good response but discontinued due to poor performance status
3	Lung AD	T810S, amplification	***EGFR*** R429S	Carboplatin	1. No new suspicious pulmonary nodules or masses.
					2. Stable upper mediastinal right paratracheal soft tissue mass at the site of prior resection.
				Paclitaxel	1. No new suspicious pulmonary nodules or masses.
					2. Stable upper mediastinal right paratracheal soft tissue mass at the site of prior resection.
4	NSCLC (NOS)	S80G		Carboplatin	Unknown
				Gemcitabine	Unknown
5	Lung AD	A848T, E886K	***EGFR*** R1068*, P518L; ***KRAS*** G10R, K169N	Carboplatin	1. No significant interval change in the large necrotic right anterior mediastinal mass with extension into the right hilum and right chest wall and sternal/pericardial/SVC invasion.
					2. Mild interval improvement in number of pulmonary nodules, specifically in the right upper lobe.
					3. Stable retroperitoneal lymphadenopathy.
					4. Nonspecific sclerotic focus in vertebral body of T3.
				Paclitaxel	1. No significant interval change in the large necrotic right anterior mediastinal mass with extension into the right hilum and right chest wall and sternal/pericardial/SVC invasion.
					2. Mild interval improvement in number of pulmonary nodules, specifically in the right upper lobe.
					3. Stable retroperitoneal lymphadenopathy. 4. Nonspecific sclerotic focus in vertebral body of T3.
6	Lung AD	E366*		CALGB 30303, Phase II IRB 13724 A trial of Docetaxel and Cisplatin	Unknown
				Carboplatin	Marked improvement of disease.
7	Lung AD	R420Q	***KRAS*** G12C	No Chemotherapy/Trial	
8	NSCLC (NOS)	K54E		Carboplatin	1. Good response.
					2. Interval decrease in size of left upper lobe mass and left hilar/subsegmental lymph node.
					3. Left upper lobe mass is now mostly cavitary.
				Paclitaxel	1. Good response.
					2. Interval decrease in size of left upper lobe mass and left hilar/subsegmental lymph node.
					3. Left upper lobe mass is now mostly cavitary.
9	NSCLC (NOS)	A757T	***KRAS*** amplification, G12V	Cisplatin	Clinically no evidence of disease
				Docetaxel	Clinically no evidence of disease
				Carboplatin	Response in some areas, but progression in right kidney (mixed response?)
				Gemcitabine	Response in some areas, but progression in right kidney (mixed response?)
10	Lung AD	Amplification	***EGFR*** L858R	Carboplatin	1. No evidence of acute pulmonary embolus.
					2. New geographic areas of groundglass opacities in the right lung.
					3. Differential diagnosis includes drug toxicity, atypical infection, and hemorrhage.
					4. Stable left lingular mass, sclerotic osseous foci, and metastatic hepatic lesions.
				Paclitaxel	1. No evidence of acute pulmonary embolus.
					2. New geographic areas of groundglass opacities in the right lung.
					3. Differential diagnosis includes drug toxicity, atypical infection, and hemorrhage.
					4. Stable left lingular mass, sclerotic osseous foci, and metastatic hepatic lesions.
				Erlotinib	Good response but discontinued due to poor performance status

AD: adenocarcinoma; NOS: not otherwise specified.

### *CBL* knockdown cells increase tumor metastasis and inhibit tumor growth of MET inhibitor PHA665752 treatment *in vivo*

**Figure 7:**
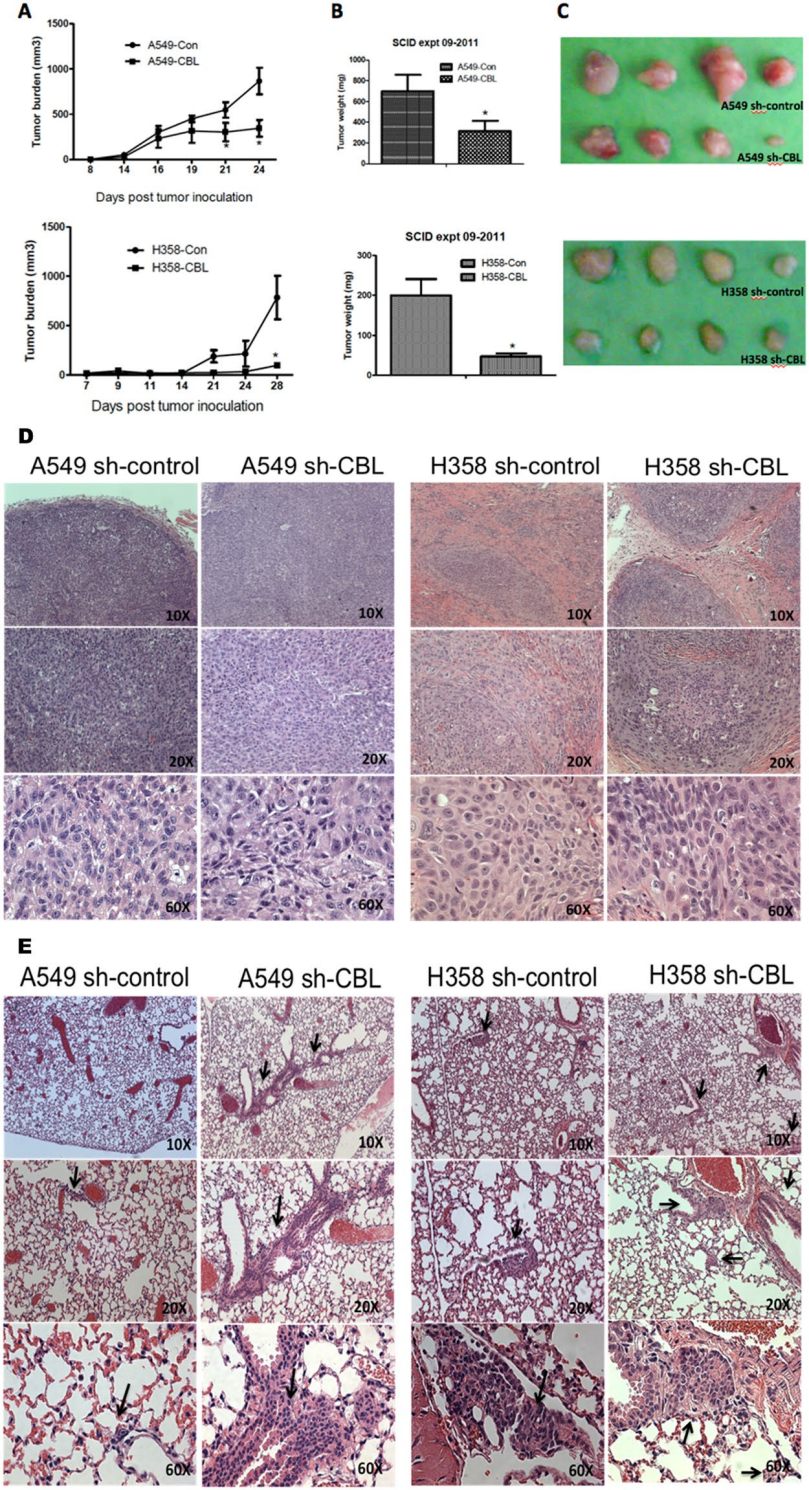
Tumor growth of *CBL* knockdown cells in mouse xenograft model. **(A)** Tumor was assessed three times a week. sh-control (con) of A549 and H358 cells showed more rapid growth of tumor than sh-CBL (CBL) cells. **(B)** Tumor weight of sh-CBL groups in A549 and H358 cells showed a significant difference compared with sh-control (* p < 0.05). **(C)** Photograph of tumors from 4 mice of sh-control and sh-CBL groups in A549 and H358 cells. **(D)** H&E staining of primary tumors from sh-control and sh-CBL groups in A549 and H358 cells at 10x, 20x, and 60x magnifications. **(E)** Metastasis study of *CBL* knockdown cells *in vivo*. H&E staining of lung tumors showed more tumor metastasis results by pathologist’s reading from the subcutaneous injection site to the lung in both A549 and H358 sh-CBL cells than in sh-control cells. Metastatic tumors shown by black arrows.

We investigated whether *CBL* knockdown affects tumor growth *in vivo* using A549 sh-CBL and H358 sh-CBL cells in mouse xenograft studies. Both A549 sh-CBL and H358 sh-CBL animals showed less tumor growth than the sh-control animals (Fig. 7A–C). However, haematoxylin and eosin (H & E) stain of lung sections showed both A549 sh-CBL and H358 sh-CBL animals had more tumor colonies in the lung than sh-control groups (Fig. 7D and E).

**Figure 8:**
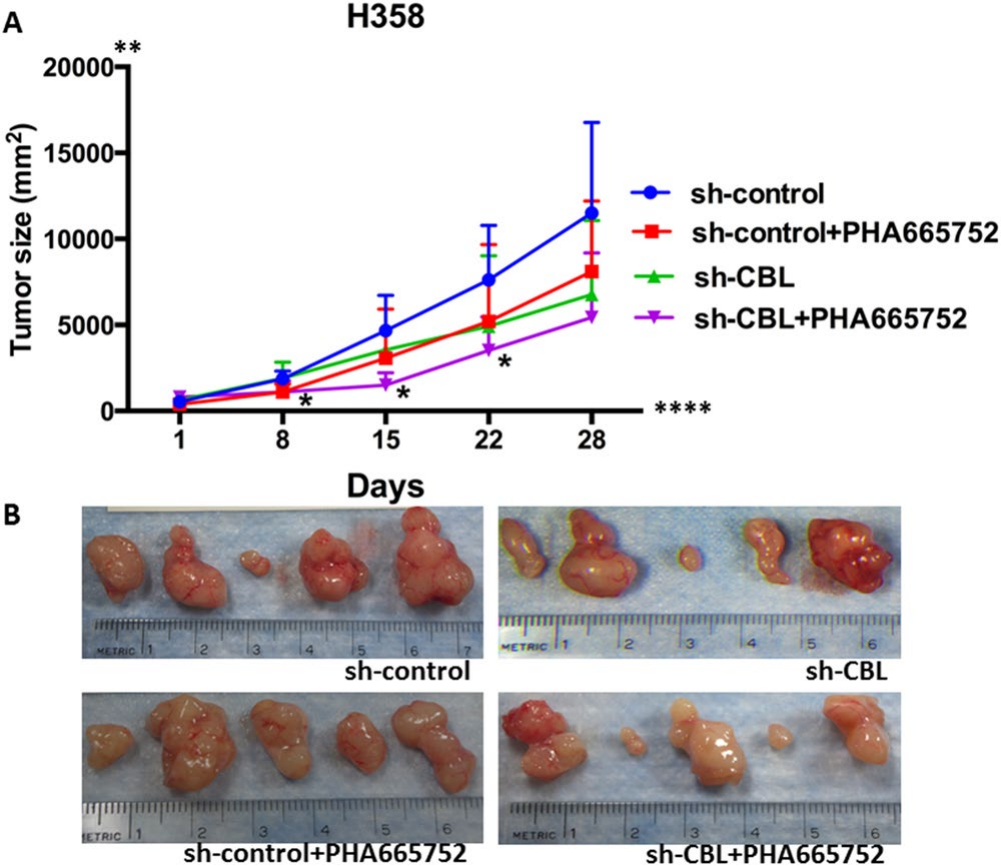
Tumor inhibition by MET inhibitor PHA665752 *in vivo*. **(A)** Tumor from sh-CBL, sh-CBL treated with PHA665752, and sh-control treated with PHA665752 groups showed growth inhibition compared with the sh-control group. *p < 0.05 **(B)** Photographs of tumors from 5 mice of each group: sh-control, sh-control treated with PHA665752, sh-CBL, and sh-CBL treated with PHA665752.

To investigate whether *CBL* can be a positive indicator for *MET*-targeted therapeutics in lung cancer, H358 sh-control/sh-CBL xenografts were treated with MET inhibitor PHA665752. The results showed that the lungs of animals in the H358 sh-CBL group had more tumor inhibition than the lungs of animals in the sh-control group. Furthermore, no tumor metastasis in the lung was found in animals in the sh-CBL group with PHA665752 treatment (Fig. 8).

The role *CBL* plays in lung cancer tumorigenesis remains unclear. From the year of 2012 to 2016, 189 NSCLC patients were enrolled in next generation sequencing platforms (FoundationONE, Foundation Medicine, Cambridge, MA) at The University of Chicago Medicine lung cancer program. We have found that 5.3% (10/189) of patients have *CBL* alterations (Table 2), which is very similar to our previous finding^13^. In FoundationONE’s report, given the mutation rates in *CBL* as well as alterations in RTKs such as *EGFR*, *MET*, or *KRAS*, it is likely that their combined effect could alter the patient treatment outcome and also be synergistic in promoting tumorigenesis. In addition, the report from the University of Chicago’s TORP database showed patients who had *CBL* and *EGFR* alterations had a poor outcome in response to EGFR treatment. This study was performed to show an alternative therapeutic strategy in lung cancer.

In the current study, immunoblotting and PamGene platforms were utilized to investigate the effective target genes of representative *CBL* Mts, S80N/H94Y double mutation, Q249E, V391I, and W802* in lung cancer. Relative to *CBL* WT transfected cells, *CBL* Mt cells had increased MET expression; *CBL* shRNA knockdown cells also showed higher MET protein expression than control cells.

*MET* is one of the “driver” genes amplified or mutated in lung cancer^15, 16, 20^. MET enhances the tumor growth and is mutated and overexpressed in many solid tumors^21^. *MET* signaling pathway activation leads to cell proliferation, survival, wound healing, motility, angiogenesis, tissue regeneration, scattering, and morphogenesis^20^. Studies have shown the *MET* mutation occurs on specific domains, e.g. juxtamembrane domain, which decreases the binding ability of CBL E3-ligase^22^. Previously, RTK overexpression and mutation were identified in many solid tumors. Recognizing oncogenic mutations of specific carcinomas allows clinicians to stop tumorigenic mechanisms by using novel targeted therapies. Unlike cytotoxic strategies, targeted therapies are often cytostatic whereas standard chemotherapy agents are cytotoxic. RTK signaling plays a role in frequent genetic aberrations in cell proliferation, tumorigenesis, metastasis, and angiogenesis; these important roles make RTK signaling an attractive target for anti-cancer therapies^23, 24^. The goal of this study was divided into three parts: first, to investigate whether MET is the target of CBL in lung cancer; second, to investigate whether *CBL* Mts affect the sensitivity of cancer cells to specific cancer therapeutics; and third, to study the response of MET inhibitor SU11274 when treated in A549 *CBL* isogenic cells. The results in A549 *CBL* isogenic cells showed the empty vector control and A549 *CBL* Mts S80N/H94Y, Q249E, V391I, and W802* cells that have very low expression of CBL and high expression of MET were more sensitive in MET inhibitor SU11274 treatment than *CBL* WT. Interestingly, there is no difference with erlotinib treatment (data not shown). According to the results shown in figure 1, *CBL* Mts decreased the ubiquitination of MET relative to *CBL* WT cells. This decreased ubiquitination brought about more MET protein expression in *CBL* Mts cells, suggesting that *CBL* Mts cells had more sensitivity in MET inhibitor SU11274 but not with erlotinib. In Table 2 we showed 10 *CBL* alteration NSCLC patients’ treatment outcome, Two out of ten patients had *EGFR* L858R/T790M mutations and had bad outcome after erlotinib treatment. Many studies have shown *EGFR* L858R/T790M cause drug resistance and poor response to gefitinib or erlotinib^25–27^. Later, we showed H1975 *EGFR* L858R/T790M mutation cells with *CBL* knockdown were sensitive with MET inhibitor SU11274 treatment but not with erlotinib. The investigation here showed a new strategy of treatment for *EGFR* L858R/T790M mutation patients who also have *CBL* alterations.

Since the loss of *CBL* (sh-CBL) and *CBL* Mts result in loss of function of CBL, we used shRNA knockdown *CBL* cells of A549 and H358 to investigate tumor growth and metastasis in mouse models. High rates of tumor metastasis were found in cell lines with knockdown *CBL*. The flank tumor of the sh-CBL group, however, did not grow as much as expected suggesting knockdown *CBL* changes the tumor phenotype and increases tumor’s ability to metastasize. The tumor cell no longer remains confined to the flank moves to distant sites. However, with MET inhibitor PHA665752 treatment, the sh-CBL group not only showed more tumor inhibition than sh-control group but also showed no metastasis in the lung after treatment. This metastatic model is more like a migration model from the subcutaneous site to the lung on cell migration *in vitro*. It is possible that there could be other mechanisms besides CBL/MET interactions that could lead to the *in vivo* tumor growth decrease with sh-CBL. What is important to note is that with sh-CBL, and MET small molecule inhibition, there is reduces the *in vivo* tumor growth beyond just sh-CBL. Further studies need to be performed to investigate the mechanism of metastasis and the relative paucity of growth at the original site of the tumor. And, that in the future, we should study the role of *CBL* as a potential predictive biomarker in anti-MET therapeutics.

In summary, this study showed that MET is regulated by CBL. The cell viability and motility results showed *CBL* Mts and sh-CBL cells are more sensitive than *CBL* WT cells to MET inhibitor SU11274. The increased sensitivity of *CBL* mutants to SU11274 suggests* CBL* mutants have higher expression of MET protein. Although MET expression in sh-CBL cells (Fig. 6) did not show dramatically different than sh-control cells, we suggest that those cells did not have 100% knockdown efficiency. Thus, MET expression was affected by endogenous CBL. As figure 1 shows, knockdown* CBL* does increase MET expression. This result can lead us to think about alternative therapeutic strategies in lung cancer patients who failed EGFR target therapy. The results of PamGene analysis indicate that MET is involved in *CBL* cell signaling transduction, especially in *CBL*-W802* cells, which are more sensitive to SU11274 compared to *CBL* WT treated with SU11274. Moreover, we noticed from kinome analysis results that Eph family (e.g. EPHA2) was also involved in *CBL* regulation. Some studies suggest that ligand stimulation induces CBL phosphorylation and regulates Eph receptors degradation^28, 29^. Other studies have shown that EPHA2 internalized by ligand-mediated stimulation and then degraded by CBL^30–33^. In our other study, EPHA2 and its ligand ephrin A1 showed overexpression, but phosphorylated EPHA2 showed low-expression in NSCLC (Data not shown). This suggests CBL plays an important but unclear role in EPHA2 degradation. In conclusion, *CBL* gene status could be a potential target and indicator for MET inhibitors and future RTK regulation investigations, especially in those that will look into whether *CBL* should be evaluated before RTK inhibitors are considered.

## Materials and Methods

### Cell culture

Human non-small-cell lung carcinoma cell lines: A549, H358, H226, and H1975 (American Type Culture Collection, Manassas, VA) were maintained in RPMI_1640_ media supplemented with 10% fetal bovine serum (FBS), 100 units/ml of penicillin, and 100 µg/ml of streptomycin (Invitrogen, Carlsbad, CA). Cells were cultured at 37 °C in a humidified incubator containing 5% CO_2_.

### *CBL* knockdown

1 × 10^5^ A549, H358, H226, and H1975 cells per well were seeded in 6-well plates and infected the following day with *CBL* lentiviral shRNA constructs (MISSION lentiviral transduction particles, Sigma-Aldrich, St. Louis, MO) per manufacturer’s instructions. To generate stable *CBL* knockdown cell lines (sh-CBL), steps were performed as described in previous work^13^.

### *CBL* constructs and transfection

A549 *CBL* knockdown cells (A549 sh-CBL) were generated as described above. Plasmid DNA of *CBL* WT and four *CBL* Mts S80N/H94Y (SH), Q249E (Q), V391I (V), and W802* (W*) were previously generated^13^. These constructs were cloned into pLenti6.3/V5-TOPO vector and transfected into A549 sh-CBL cells using Fugene HD reagent (Roche, Nutley, NJ) according to the manufacturer’s instructions. After 48 hours, cells were harvested and analyzed for CBL expression. Cells were cultured in 5 mg/ml of blasticidin (Invitrogen) for stable selection.

### Cell viability assay

A549 *CBL* isogenic cells were transfected with *CBL* WT and Mts as described above. Forty-eight hours after transfection, cells were harvested and re-seeded at 5 × 10^4^ cells/well in a 24-well culture plate. After 24 hours, cells were treated with 1 μM of MET inhibitor SU11274 for 48 hours. Cell viability was determined using Trypan Blue exclusion.

H1975 *CBL* knockdown cells were seeded 5 × 10^3^/well in a 96-well culture plate. After 24 hours, cells were treated with MET inhibitor SU11274 and EGFR inhibitor, erlotinib, for 24, 48, and 72 hours. Cell viability was determined using Calcein-AM exclusion.

### Ubiquitin ligase activity

This assay was performed as described in previous work^34^. Proteins were collected and blotted with anti-MET, anti-Ubiquitin, anti-HA, and anti-β-actin antibodies.

### Immunoblotting

Cells were collected at 48 hours after transfection and performed a standard steps as previously described^13^. Antibodies were used at the following dilutions and obtained from Santa Cruz Biotechnologies (CBL, 1:500; MET, 1:1000; EGFR, 1:1000; Ubiquitin, 1:1000; HA, 1:1000; and β-actin, 1:2000), Cell Signaling Technology (Danvers, MA) (p-EPHA2 Y772, 1:1000; p-CD79A Y182, 1:1000), Abcam (Cambridge, MA) (p-p85 Y607, 1:500), and OriGene Technologies (Rockville, MD) (p-PECAM Y713, 1:500). The immunoblotting results were quantified by ImageJ and Quantity Once software (Bio-Rad, Hercules, CA).

### Wound healing assay

5 × 10^4^ H358 sh-CBL cells transfected as described in the transfection assay and/or treated with 1 μM of SU11274 were seeded in a wound healing assay insert (ibidi, Fitchburg, WI) for 24 hours. After removing the insert, the cells were then gently washed with 1X phosphate-buffered saline (PBS) to remove cellular debris and the media was replaced. Photographs of the wound region were taken every 3 hours until 52 hours had passed. The images were analyzed using the TScratch software (Computational Science and Engineering Laboratory, ETH Zurich, Switzerland). The wound closure at each time point was quantified and normalized to 0 hr.

### Soft agar colony formation assay

To investigate the potential of *CBL* cells tumorigenesis, 5 × 10^4^ viable H358 and H226 sh-control and sh-CBL cells per well were seeded in soft agar in 6-well plates. The base layer was 0.6% agar in 1X RPMI1640 medium and the top layer was 0.4% agar in 1X RPMI1640 medium. Cells were mixed in the top layer and grew for 4 weeks at 37 °C in a humidified atmosphere containing 5% CO_2_. Viable colonies were photographed and counted using ImageJ software (http://rsbweb.nih.gov/ij/). In the drug treatment study, H226 sh-control and sh-CBL cells were pretreated with 2 μM MET inhibitor SU11274 for 48 hours and 5 × 10^4^ viable cells per well were plated in soft agar in 6-well plates, following the same experimental procedure described above.

### PamGene technology and analysis

PamGene technology uses the PamChip® Tyrosine Kinase Array of the phosphorylation of peptides spotted onto a 3-dimensional porous well of a 4-array chip produced and commercialized by PamGene (s-Hertogenbosch, The Netherlands). There are 144 peptides on each array. PamGene measures the activity of kinases in whole cell lysates. Evaluating the effects of CBL on the phosphor-kinome was performed as previously described by the PamGene platform^35, 36^.

The kinase activity of A549 *CBL* isogenic cells with and without MET inhibitor SU11274 was monitored. In brief, cells were treated with SU11274, harvested and lysed in Mammalian Extraction Buffer (M-PER, Pierce) containing phosphatase and protease inhibitors (HALT, Pierce). Five μl of the lysis solution was pipetted into a reaction mixture composed of 1X ABL buffer (New England Biolabs), 0.1% Bovine Serum Albumin, 100 μM ATP, 20 μg/ml phosphor-tyrosine antibody in a total volume of 40 μl. PamChips arrays were blocked with 0.2% BSA prior to loading the samples. After loading the reaction mixtures onto the Pamchip arrays real time data for the kinase activity were obtained by measuring fluorescence of the bound anti-phospho-tyrosine antibody after each of the 5 cycles. Image quantification and data processing were conducted with dedicated Pamgene software Evolve and BioNavigator (PamGene). The peptides that were significantly differentially affected peptides and signaling pathways were analyzed using Ingenuity Pathway Analysis (IPA) (Redwood City, CA).

### Tumor growth and metastasis analysis *in vivo*

A459 and H358 sh-control and sh-CBL cells were cultured in RPMI_1640_ medium with 10% FBS and 1% penicillin/streptomycin at 37 °C in a humidified incubator containing 5% CO_2_. Once the cells reached a 60% of density, cells were trypsinized and harvested in HBSS. Eight-week-old SCID/Beige mice were used for this study. Viable cells (5 × 10^6^) were suspended in 0.1 ml sterile PBS and were injected into the right suprascapular region of each mouse. Tumor burden was measured using bisecting diameters with calipers. Tumor volume of each group was plotted against time. Tumor volume was calculated using the formula 0.4ab^2^ (a = large diameter and b = small diameter). Tumor and perfused lung samples were fixed in 4% formalin, embedded in paraffin, and stained with hematoxylin and eosin (H & E). Once the animals were sacrificed, the tumors weights were determined.

For drug inhibition investigation, female BALB/c nude mice, age 5–6 weeks, were used after obtaining appropriate Institutional Review Board (IRB) approval and raised in a pathogen-free environment. H358 sh-control and sh-CBL cells (5 × 10^6^) in a volume of 200 μl were implanted subcutaneously into the mice. H358 sh-control and sh-CBL xenograft were treated with MET inhibitor PHA665752 (150 μg/50 μl of 2% DMSO) by intraperitoneal (i.p.) continually for 8 days after tumor nodule size reach to 50 mm^3^. Tumors were measured with calipers and collected.

All experiments and animal care were performed in accordance with Institutional Animal Care and Use Committees (IACUC) animal care guidelines and the University of Chicago Automating University-wide Research Administration (AURA) Institutional Biosafety Committee (IBC) approval.

### Clinical data interpretation

All the clinical data and the associated molecular data presented in this project were queried from the University of Chicago’s Thoracic Oncology Research Program (TORP) database. The TORP database is comprised of four component databases: the Microsoft Access database, the Velos database, the REDCap database, and the Microsoft Excel database. For this project, we mainly utilized the Access database for acquiring the necessary data. The Access database serves as the central repository for clinical, demographical, research, and molecular data.

In the process of obtaining the clinical and molecular data, patients were first consented onto the 9571 and 13473 A, which are both IRB approved protocols. These two protocols authorize the use of retrospectively and/or prospectively collected of biospecimens, as well as associated clinical, demographical, and molecular data. After patients were consented, patient data were extracted from electronic medical health records and transferred into the Access database by the TORP’s data manager. The data manager then performed querying functions in the Access database to acquire the necessary data for the project and performed in accordance with relevant guidelines and regulations.

### Statistical analysis

Experiments involving repeated measurements over time were analyzed using repeated measures analysis of variance (ANOVA) with the Greenhouse-Geisser adjustment. Group comparisons were done by ANOVA with the Sidak adjustment. Analyses were conducted using STATA (v10.1) software (Stata Corporation, College Station, TX).

## Electronic supplementary material

Supplementary Material
