# Supplementary material for: Differential responsiveness of MET inhibition in non-small-cell lung cancer with altered CBL
Source: Sci Rep. 2017 Aug 23;7:9192. doi: 10.1038/s41598-017-09078-4 (PMC5569108; doi:10.1038/s41598-017-09078-4)
Supplement: Supplementary file 1 — Supplementary Material [file 41598_2017_9078_MOESM1_ESM.pdf]

Differential responsiveness of MET inhibition in non-small-cell lung cancer with altered CBL

Yi-Hung Carol Tan[1], Tamara Mirzapoiazova[2], Brian M. Won[1], Li Zhu[3], Minu K. Srivastava[3], Everett E. Vokes[1], Aliya N. Husain[4], Surinder K. Batra[5], Sherven Sharma[3], and Ravi Salgia[2*]

[1]Department of Medicine, Section of Hematology/ Oncology, The University of Chicago Medicine and Biologic Sciences, Chicago, IL, USA.

[2]Department of Medical Oncology and Therapeutic Research, City of Hope, Duarte, CA, USA.

[3]Department of Medicine, University of California Los Angeles, Los Angeles, CA, USA.

[4]Department of Pathology, The University of Chicago Medicine and Biologic Sciences, Chicago, IL, USA.

[5]Department of Biochemistry and Molecular Biology, University of Nebraska Colle of Medicine, Fred and Pamela Buffett Cancer Center, University of Nebraska Medical Center, Omaha, NE, USA.

SUPPLEMENTARY FIGURES

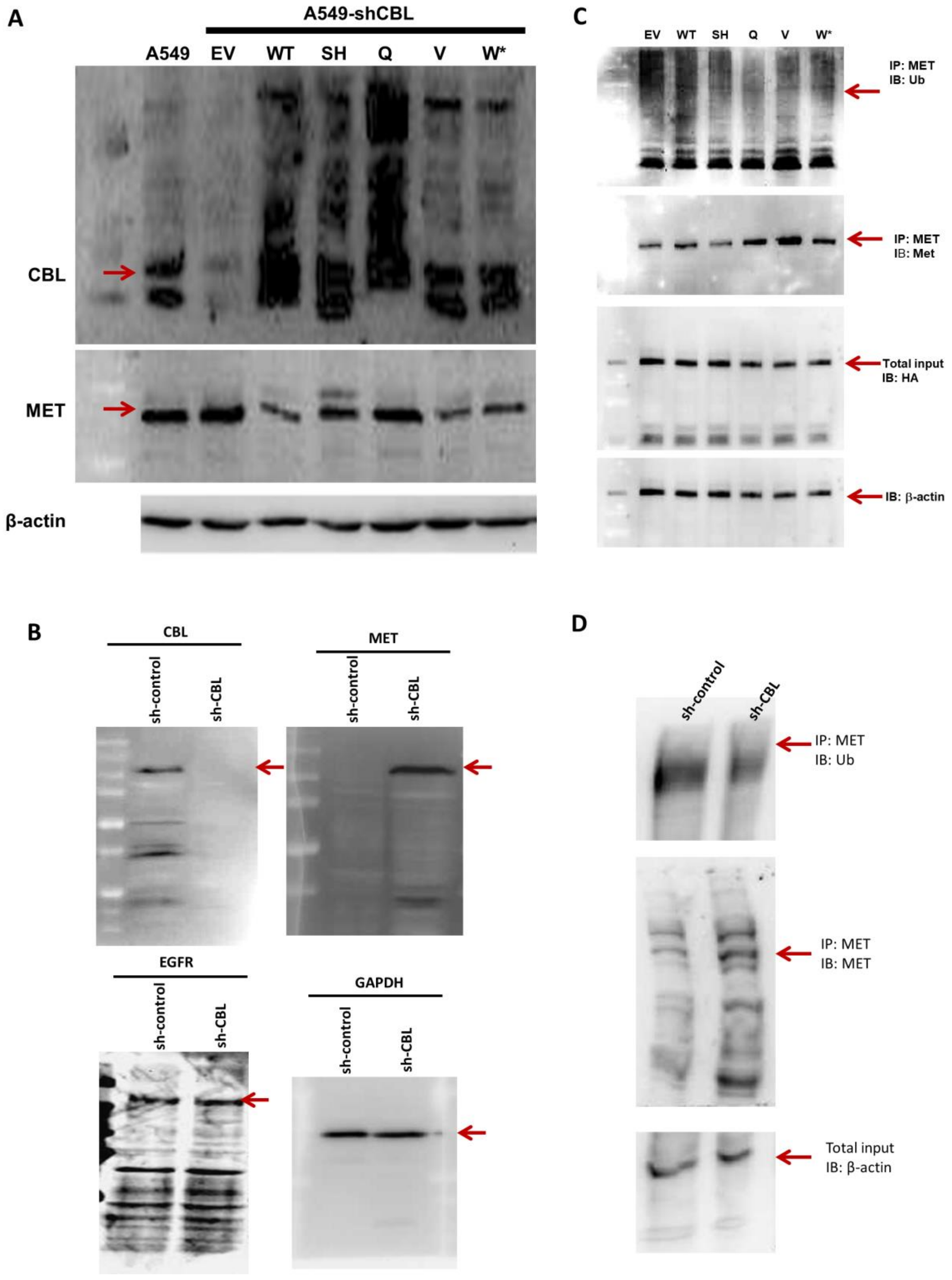

Supplementary Figure 1. Full length blots of **figure 1. Ubiquitination and expression analysis of various *CBL* mutants. (A)** A549 shRNA knockdown *CBL* cells were transiently transfected with various *CBL* mutants (SH: S80N/H94Y, Q: Q249E, V: V391I, W*: W802*) and

wild-type (WT). MET protein showed low expression in *CBL* WT and high expression in *CBL* Mts isogenic cells. Protein expression was quantified and indicated with the fold change numbers shown below each immunoblot in comparison with loading control β-actin. **(B)** MET and EGFR expression of H358 sh-control and sh-CBL. MET showed higher expression in sh-CBL than sh-control cells. EGFR had no difference in sh-control and sh-CBL. **(C)** A549 cells transiently transfected with empty vector (EV) or *CBL* WT and Mts (SH: S80N/H94Y, Q: Q249E, V: V391I, W*: W802*). Whole cell lysates were IP with anti-MET antibody and IB with anti-Ub antibody. IB with anti-HA antibody for transfection efficiency and β-actin for loading control of the IP. The results showed the ubiquitination of MET were decreased in A549 cells that transiently expressed CBL mutants relative to CBL WT cells. **(D)** H358 sh-Control and sh-CBL cell lysates were IP with anti-MET antibody and IB with anti-Ub antibody β-actin for loading control of the IP. The results showed the ubiquitination of MET were decreased in sh-CBL cells relative to sh-control cells. Each protein lysates of separated blot of were collected in the same time period for and the lysates were loaded in one gel per antibody staining.

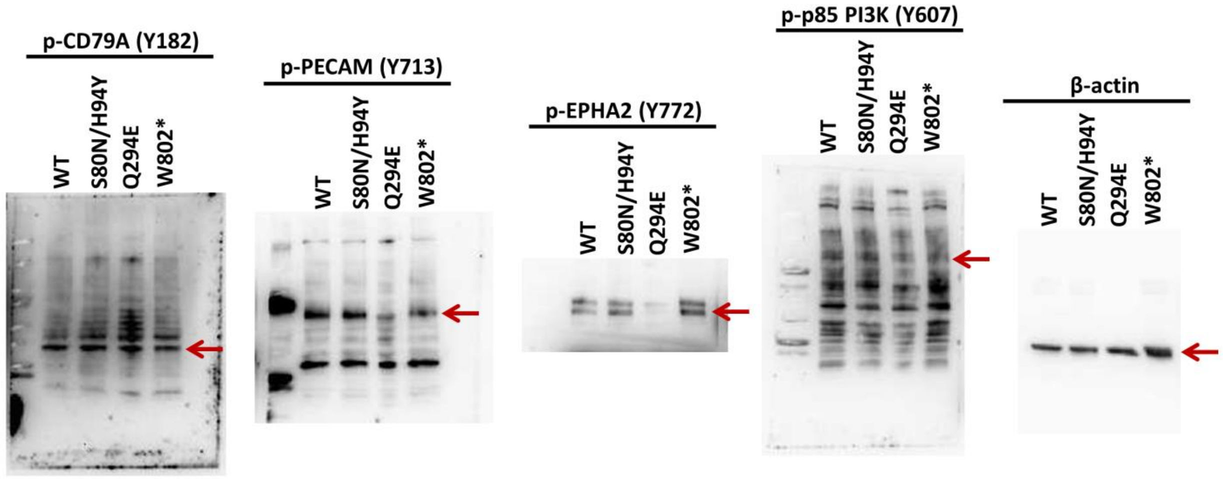

Supplementary Figure 2. Full length blots of **figure 5B.** The protein expression of common targets in three CBL mutations identified from Pamgene was validated by immunoblotting. Protein expression was quantified and indicated with the fold change numbers shown below each immunoblot in comparison with WT. Each protein lysates of separated blot of were collected in the same time period for and the lysates were loaded in one gel per antibody staining.

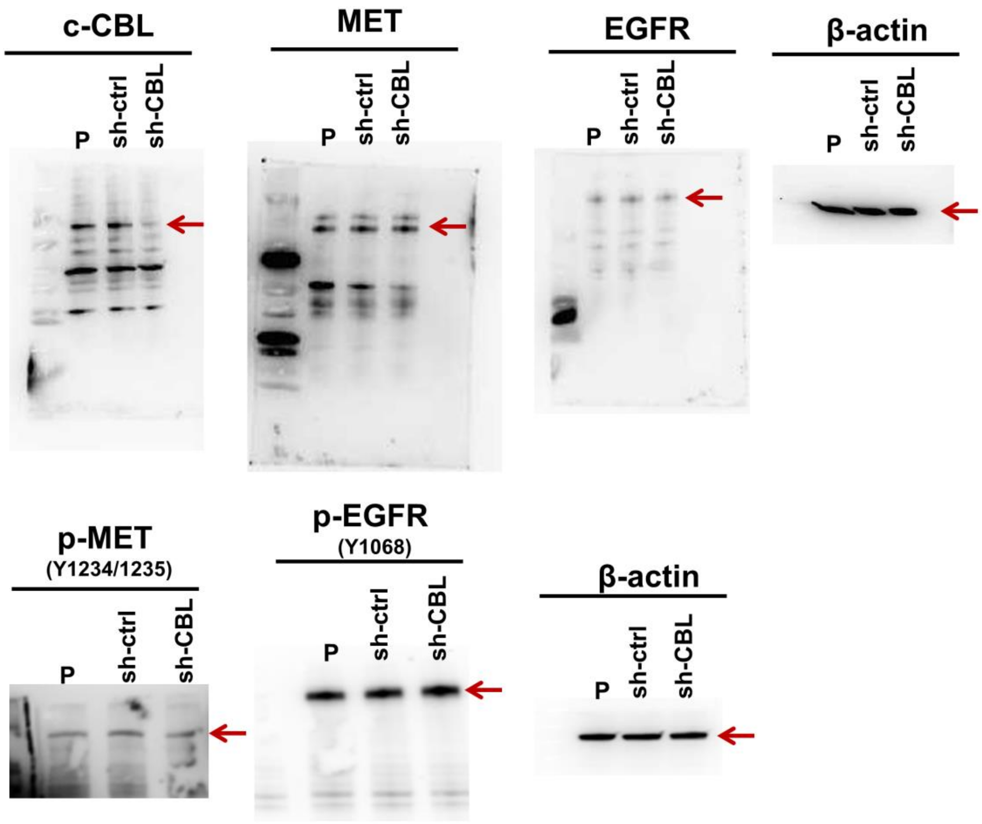

Supplementary Figure 3. Full length blots of **figure 6A.** CBL, MET, p-MET, EGFR, and p-EGFR protein expression in H1975 CBL knockdown cells. Protein expression was quantified and indicated with the fold change numbers shown below each immunoblot in comparison with parental H1975 cells. (p: parental, c: sh-control).
